# Crystal structures of potassium tri­fluorido­(4-meth­oxy­phen­yl)borate and potassium tri­fluorido(4-fluoro­phen­yl)borate

**DOI:** 10.1107/S1600536814009684

**Published:** 2014-07-19

**Authors:** William T. A. Harrison, James L. Wardell

**Affiliations:** a Department of Chemistry, University of Aberdeen, Meston Walk, Aberdeen AB24 3UE, Scotland; b FioCruz-Fundação Oswaldo Cruz, Instituto de Tecnologia em Fármacos-Far-Manguinhos, Rua Sizenando Nabuco, 100, Manguinhos, 21041-250 Rio de Janeiro, RJ, Brazil

**Keywords:** crystal structure, boron, Lewis acid, layered structure

## Abstract

The title compounds, K^+^·C_7_H_7_BF_3_O^−^, (I), and K^+^·C_6_H_4_BF_4_^−^, (II), are mol­ecular salts containing *para*-substituted phenyl­tri­fluorido­borate anions. In each compound, the B atom adopts a distorted tetra­hedral BCF_3_ geometry. Despite their different compositions and space groups, the irregular KF_8_ coordination polyhedra of the potassium cations in the structures are almost identical. These polyhedra share faces and edges, generating infinite (010) layers in (I) and infinite (001) layers in (II). In (I), adjacent layers are stacked in an *AAA*… fashion, whereas in (II), they are stacked in an *ABAB*… sequence.

## Abstract 2

Despite their different compositions and space groups, the irregular KF_8_ coordination polyhedra of the potassium cations in these structures are almost identical. The layer stacking sequences are *AAA*… in the *p*-methoxy compound and *ABAB*… in the *p*-fluoro compound.

## Chemical context   

The phenyl­tri­fluorido­borate anion is an inter­esting inter­mediate species between the well-known tetra­fluorido­borate (BF_4_^−^) and tetra­phenyl­borate [B(C_6_H_5_)_4_^−^] ions (Conole *et al.*, 1995 ▸) and may serve as a bulky charge-balancing anion (Quach *et al.*, 2001 ▸; Fei *et al.*, 2010 ▸). As part of our studies in this area, we now describe the syntheses and structures of the *para*-substituted phenyl­tri­fluorido­borate salts K^+^C_7_H_7_BF_3_O^−^ (I) and K^+^C_6_H_4_BF_4_^−^ (II).
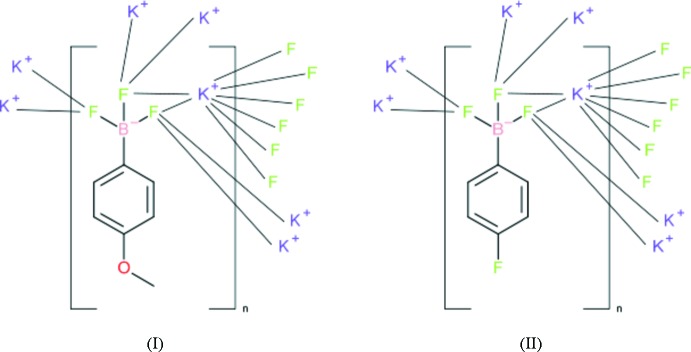


## Structural commentary   

**Figure 1:**
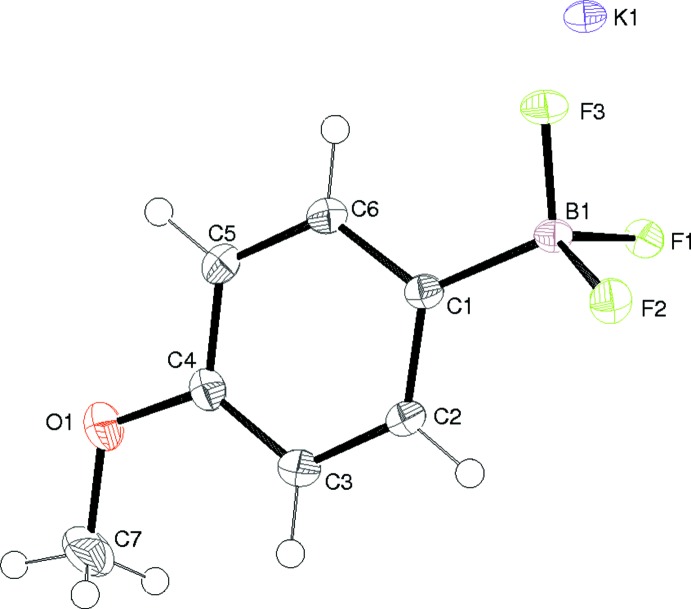
The asymmetric unit of (I) showing 50% displacement ellipsoids.

Compound (I) comprises one cation and one anion in the asymmetric unit (Fig. 1 ▸). In the anion, the C7 atom of the meth­oxy group is close to coplanar with the benzene ring [displacement = 0.048 (2) Å]. The B atom adopts its expected tetra­hedral BF_3_C geometry (Conole *et al.*, 1995 ▸) and the C1—B1 bond length of 1.5987 (18) Å is consistent with previous data (Quach *et al.*, 2001 ▸). One of the B—F bonds (to F1) in (I) is notably longer than the other two, which might reflect the different modes of coordination of the fluorine atoms to the potassium ions. The F—B—F bond angles (mean = 105.7°) are significantly smaller than the C—B—F angles (mean = 113.0°). F1 is displaced by −1.427 (2) Å from the plane of the benzene ring and F2 and F3 are displaced in the opposite sense, by 0.715 (2) and 0.252 (2) Å, respectively.

**Figure 2:**
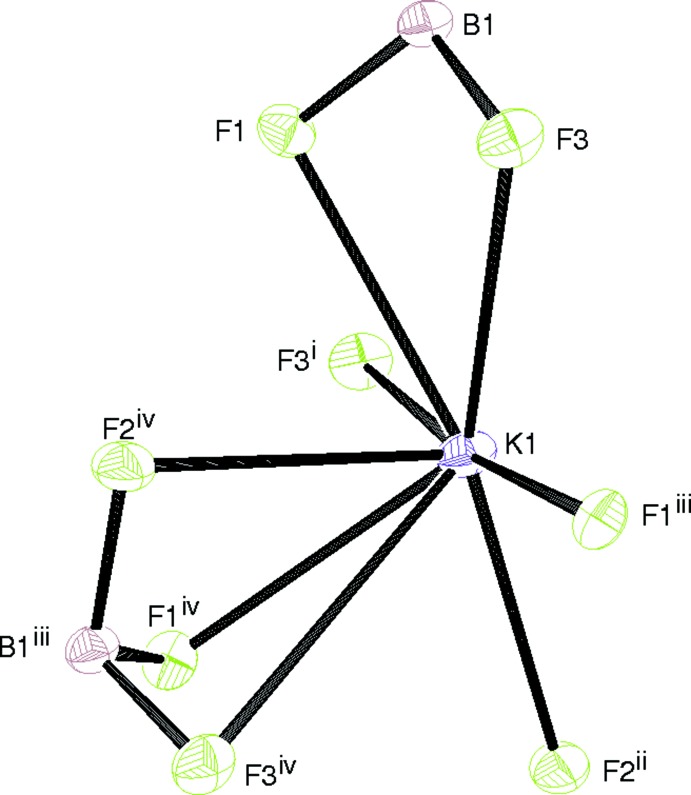
The coordination of the potassium ion in (I). See Table 1 ▸ for symmetry codes.

The potassium ion in (I) is coordinated by eight fluorine atoms, with one of the K—F bonds substanti­ally longer than the others (Table 1 ▸): the next-nearest F atom is over 4 Å distant. The coordination geometry of the K^+^ ion, which arises from one tridentate, one bidentate and three monodentate BF_3_^−^ groups, is irregular and highly asymmetric (Fig. 2 ▸), with five of the F atoms forming an approximate plane and the other three (arising from one BF_3_ group) lying to one side. The metal ion is displaced by 1.00 Å from the geometric centroid of the eight F atoms. In terms of the F atoms in (I), F1 bonds to three different metal ions (mean K—F = 2.734 Å), generating a distorted FBK_3_ tetra­hedron, whereas F2 bonds to two K^+^ ions (mean K—F = 2.755 Å) in an FBK_2_ distorted T-shape. If the geometry around F3 is not merely deemed to be irregular, it could be described as an FBK_3_ trigonal-based pyramid, with the long K—F bond (Table 1 ▸) as the apex (mean K—F = 2.963 Å). The extended structure in (I) consists of (010) sheets in which the KF_8_ polyhedra share faces in the [100] direction and edges in [001]: the shortest K⋯K separation is 4.4523 (4) Å.

**Figure 3:**
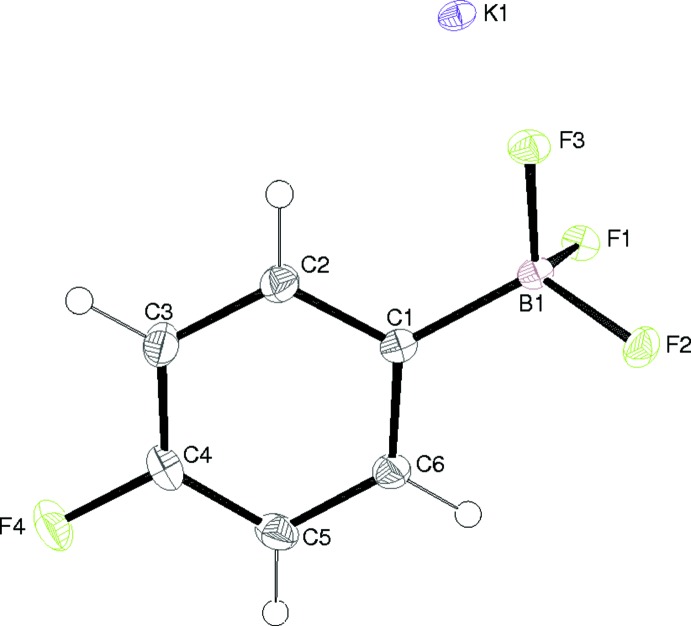
The asymmetric unit of (II) showing 50% displacement ellipsoids.

The asymmetric unit of compound (II) also consists of an ion-pair (Fig. 3 ▸). The geometry of the anion in (II) is very similar to that of the equivalent species in (I): the C1—B1 bond length is 1.590 (2) Å and the mean F—B—F and C—B—F bond angles are 105.5 and 113.2°, respectively. The displace­ments of F1, F2 and F3 from the benzene-ring plane are −1.386 (2), 0.813 (3) and 0.131 (3) Å, respectively. As seen for (I), the B1—F1 bond in (II) is noticeably longer than the B1—F2 and B1—F3 bonds.

**Figure 4:**
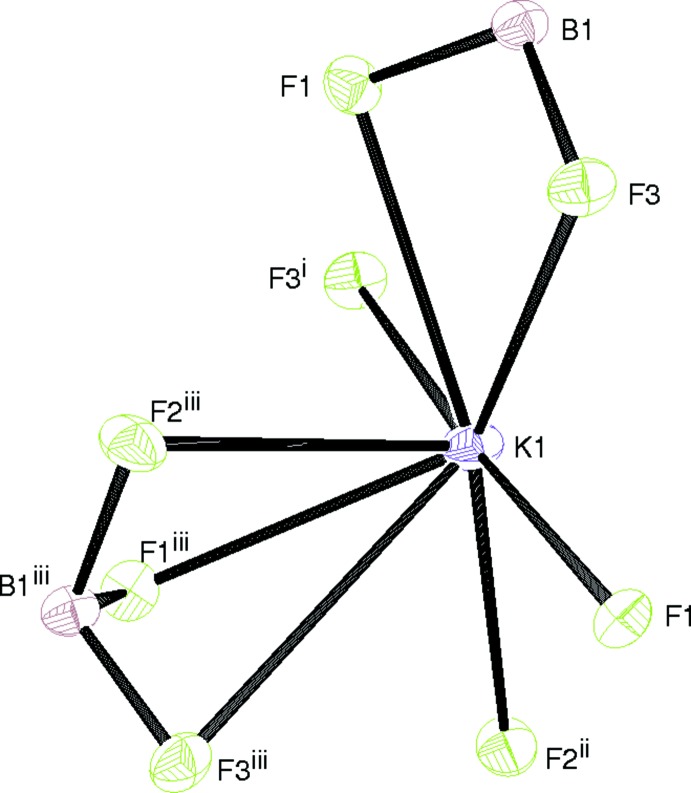
The coordination of the potassium ion in (II). See Table 2 ▸ for symmetry codes.

It is notable that the K^+^ ion in (II) adopts a very similar coordination geometry (Table 2 ▸) to the equivalent species in (I), despite the different space groups. Again, a very asymmetric KF_8_ coordination polyhedron (Fig. 4 ▸) arises from one tridentate, one bidentate and three monodentate anions; one K—F bond is much longer than the others and the potassium ion is displaced by 0.98 Å from the geometric centroid of the fluorine atoms.

The extended structure of (II) consists of (001) sheets [rather than (010) sheets, as seen in (I)] of face- and edge-sharing KF_8_ groups with the same topology as in (I): the shortest K⋯K separation is 4.4255 (5) Å.

## Supra­molecular features   

**Figure 5:**
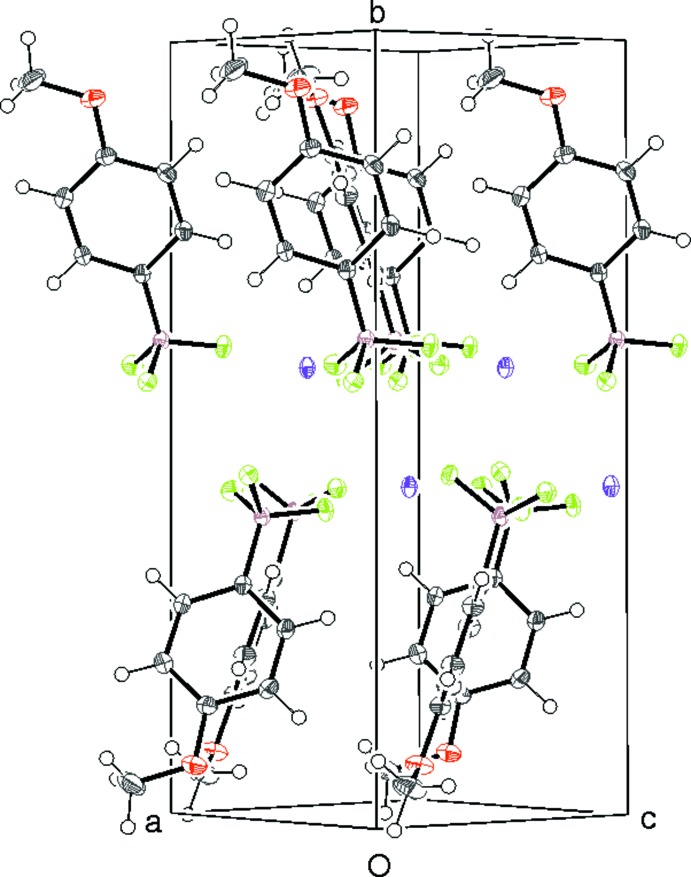
The unit-cell packing in (I) viewed approximately down [101].

In (I) the meth­oxy­phenyl groups lie roughly normal to (010). When the packing is viewed along [101] (Fig. 5 ▸), it may be seen that adjacent benzene ring planes are rotated by 90°, which facilitates the formation of a weak edge-to-face intra-sheet C—H⋯π inter­action (Table 3 ▸). An intra-sheet C2—H2⋯F2 hydrogen bond also occurs. The only possible inter-sheet inter­action in (I) is an extremely weak C—H⋯O hydrogen bond with an H⋯O separation essentially the same as the van der Waals separation of these species. The layer-stacking sequence for (I) is *AAA*….

**Figure 6:**
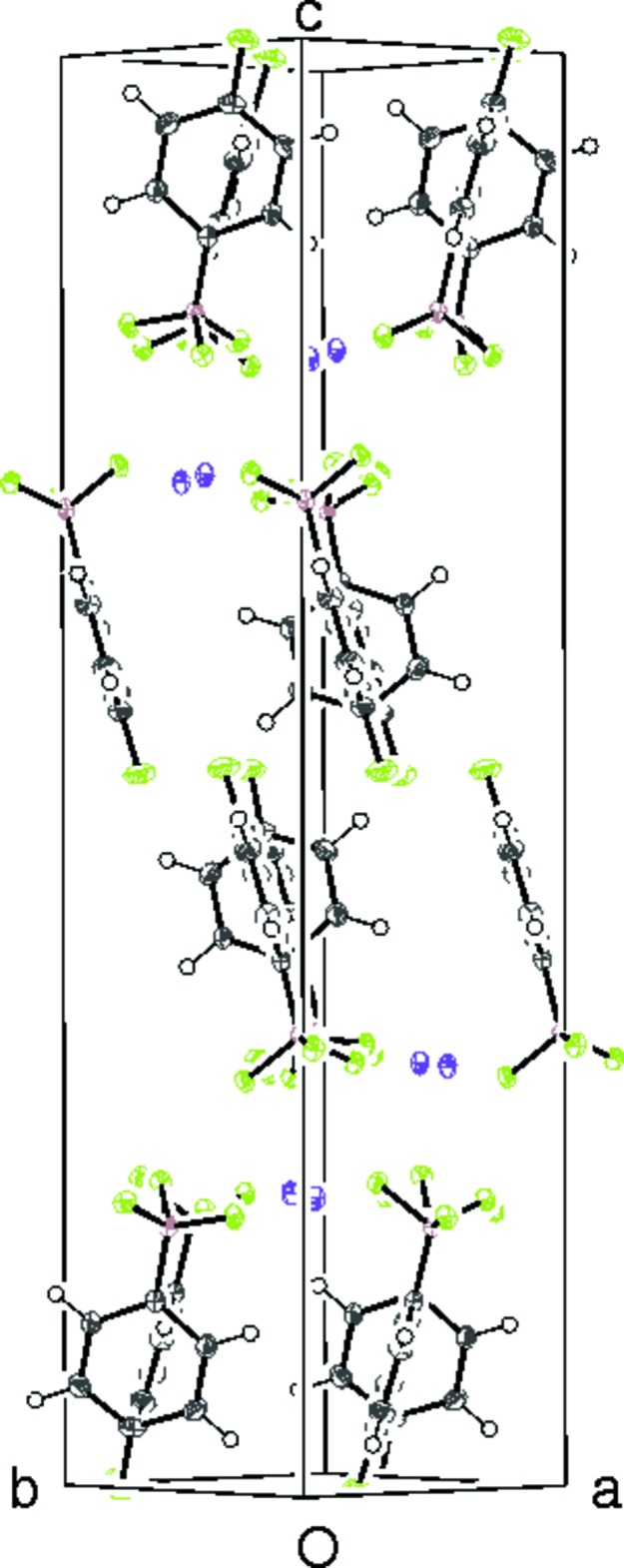
The unit-cell packing in (II) viewed approximately down [110].

When the crystal structure of (II) is viewed down [110] (Fig. 6 ▸), adjacent aromatic rings show the same 90° rotation as they do in (I), but the only directional inter­action identified is an intra­layer weak C—H⋯F hydrogen bond (Table 4 ▸) and there are no C—H⋯π inter­actions. There are no identified inter-layer inter­actions and the stacking sequence is *ABAB*….

## Database survey   

Compound (I) is closely related to K^+.^C_6_H_5_BF_3_^−^ (Conole *et al.*, 1995 ▸), (III). Compounds (I) and (III) have the same space group and a similar unit cell, extended in the *b*-axis direction for (I) to accommodate the meth­oxy group. The potassium ion in (III) has almost the same KF_8_ coordination geometry as the equivalent species in (I) and (II) described above. In (III), weak edge-to-face C—H⋯π inter­actions occur between approximately perpendicular aromatic rings, as they do in (I). As already noted, the C_6_H_5_BF_3_^−^ anion has found use as a bulky charge-balancing species (Quach *et al.*, 2001 ▸; Fei *et al.*, 2010 ▸).

## Synthesis and crystallization   

(I) and (II) were received as commercial samples from Aldrich and recrystallized from ethanol solution, yielding colourless blocks.

## Refinement   

The H atoms were placed in idealized positions (C—H = 0.95–0.98 Å) and refined as riding atoms with *U*_iso_(H) = 1.2*U*_eq_(C) or 1.5*U*_eq_(methyl C). The methyl group in (I) was allowed to rotate, but not to tip, to best fit the electron density.. Experimental details are given in Table 5 ▸.

## Supplementary Material

Crystal structure: contains datablock(s) I, II, global. DOI: 10.1107/S1600536814009684/wm0001sup1.cif

Structure factors: contains datablock(s) I. DOI: 10.1107/S1600536814009684/wm0001Isup2.hkl

Structure factors: contains datablock(s) II. DOI: 10.1107/S1600536814009684/wm0001IIsup3.hkl

CCDC references: 1004280, 1004281

Additional supporting information:  crystallographic information; 3D view; checkCIF report

## Figures and Tables

**Table 1:** Selected bond lengths (Å) for (I)

K1—F3^i^	2.6156 (10)	K1—F2^iv^	2.8885 (8)
K1—F2^ii^	2.6211 (7)	K1—F3^iv^	3.4886 (9)
K1—F1^iii^	2.6550 (10)	B1—F3	1.4162 (19)
K1—F1^iv^	2.7568 (10)	B1—F2	1.4196 (14)
K1—F3	2.7836 (8)	B1—F1	1.4403 (17)
K1—F1	2.7887 (8)		

Symmetry codes: (i) 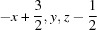; (ii) 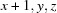; (iii) 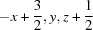; (iv) 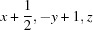.

**Table 2:** Selected bond lengths (Å) for (II)

K1—F3^i^	2.6116 (10)	K1—F2^iv^	2.8853 (10)
K1—F2^ii^	2.6159 (9)	K1—F3^iv^	3.3927 (10)
K1—F1^iii^	2.6527 (9)	B1—F2	1.4166 (17)
K1—F1^iv^	2.7612 (10)	B1—F3	1.4182 (19)
K1—F3	2.7732 (9)	B1—F1	1.4393 (18)
K1—F1	2.8050 (9)		

Symmetry codes: (i) 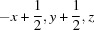; (ii) 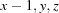; (iii) 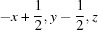; (iv) 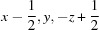.

**Table 3:** Hydrogen-bond geometry (Å, °) for (I) *Cg*1 is the centroid of the C1–C6 benzene ring.

*D*—H⋯*A*	*D*—H	H⋯*A*	*D*⋯*A*	*D*—H⋯*A*
C2—H2⋯F2^v^	0.95	2.50	3.3359 (17)	147
C7—H7a⋯O1^vi^	0.98	2.72	3.496 (1)	137
C3—H3⋯*Cg*1^v^	0.95	2.85	3.7171 (15)	152

Symmetry codes: (v) 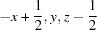; (vi) 
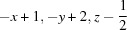
.

**Table 4:** Hydrogen-bond geometry (Å, °) for (II)

*D*—H⋯*A*	*D*—H	H⋯*A*	*D*⋯*A*	*D*—H⋯*A*
C6—H6⋯F2^v^	0.95	2.53	3.4099 (19)	154

Symmetry code: (v) 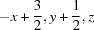.

**Table 5:** Experimental details

	(I)	(II)
Crystal data		
Chemical formula	K^+^·C_7_H_7_BF_3_O^−^	K^+^·C_6_H_4_BF_4_^−^
*M*_r_	214.04	202.00
Crystal system, space group	Orthorhombic, *P* *c* *a*2_1_	Orthorhombic, *P* *b* *c* *a*
Temperature (K)	120	100
*a*, *b*, *c* (Å)	7.1347 (2), 17.2819 (7), 7.3289 (3)	7.1317 (5), 7.3757 (5), 29.129 (2)
*V* (Å^3^)	903.66 (6)	1532.22 (18)
*Z*	4	8
Radiation type	Mo *K*α	Mo *K*α
μ (mm^−1^)	0.59	0.70
Crystal size (mm)	0.52 × 0.15 × 0.15	0.07 × 0.05 × 0.01

Data collection		
Diffractometer	Rigaku CCD	Rigaku CCD
Absorption correction	Multi-scan (*SADABS*; Bruker, 2012 ▸)	Multi-scan (*SADABS*; Bruker, 2012 ▸)
*T*_min_, *T*_max_	0.750, 0.917	0.953, 0.993
No. of measured, independent and observed [*I* > 2σ(*I*)] reflections	5789, 1833, 1822	9537, 1726, 1435
*R*_int_	0.026	0.037
(sin θ/λ)_max_ (Å^−1^)	0.649	0.649

Refinement		
*R*[*F* ^2^ > 2σ(*F* ^2^)], *wR*(*F* ^2^), *S*	0.021, 0.059, 1.10	0.027, 0.065, 1.06
No. of reflections	1833	1726
No. of parameters	120	109
No. of restraints	1	0
H-atom treatment	H-atom parameters constrained	H-atom parameters constrained
Δρ_max_, Δρ_min_ (e Å^−3^)	0.24, −0.20	0.27, −0.23
Absolute structure	Flack (1983 ▸), 712 Friedel pairs	–
Absolute structure parameter	0.02 (3)	–

Computer programs: *CrystalClear* (Rigaku, 2010 ▸), *SHELXS97* and *SHELXL97* (Sheldrick, 2008 ▸), *ORTEP-3 for Windows* (Farrugia, 2012 ▸) and *publCIF* (Westrip, 2010 ▸).

## supplementary crystallographic information

### (I) Potassium trifluorido(4-methoxyphenyl)borate Crystal data

**Table:**

K^+^·C_7_H_7_BF_3_O^−^	*D*_x_ = 1.573 Mg m^−^^3^
*M**_r_* = 214.04	Mo *K*α radiation, λ = 0.71075 Å
Orthorhombic, *P**c**a*2_1_	Cell parameters from 4830 reflections
*a* = 7.1347 (2) Å	θ = 1.2–27.5°
*b* = 17.2819 (7) Å	µ = 0.59 mm^−^^1^
*c* = 7.3289 (3) Å	*T* = 120 K
*V* = 903.66 (6) Å^3^	Block, colourless
*Z* = 4	0.52 × 0.15 × 0.15 mm
*F*(000) = 432	

### (I) Potassium trifluorido(4-methoxyphenyl)borate Data collection

**Table:**

Rigaku CCD diffractometer	1833 independent reflections
Radiation source: fine-focus sealed tube	1822 reflections with *I* > 2σ(*I*)
Graphite monochromator	*R*_int_ = 0.026
ω scans	θ_max_ = 27.5°, θ_min_ = 1.2°
Absorption correction: multi-scan (*SADABS*; Bruker, 2012)	*h* = −9→9
*T*_min_ = 0.750, *T*_max_ = 0.917	*k* = −22→20
5789 measured reflections	*l* = −8→9

### (I) Potassium trifluorido(4-methoxyphenyl)borate Refinement

**Table:**

Refinement on *F*^2^	Hydrogen site location: inferred from neighbouring sites
Least-squares matrix: full	H-atom parameters constrained
*R*[*F*^2^ > 2σ(*F*^2^)] = 0.021	*w* = 1/[σ^2^(*F*_o_^2^) + (0.035*P*)^2^ + 0.1245*P*] where *P* = (*F*_o_^2^ + 2*F*_c_^2^)/3
*wR*(*F*^2^) = 0.059	(Δ/σ)_max_ < 0.001
*S* = 1.10	Δρ_max_ = 0.24 e Å^−^^3^
1833 reflections	Δρ_min_ = −0.20 e Å^−^^3^
120 parameters	Extinction correction: *SHELXL97* (Sheldrick, 2008), Fc^*^=kFc[1+0.001xFc^2^λ^3^/sin(2θ)]^-1/4^
1 restraint	Extinction coefficient: 0.0115 (14)
Primary atom site location: structure-invariant direct methods	Absolute structure: Flack (1983), 712 Friedel pairs
Secondary atom site location: difference Fourier map	Absolute structure parameter: 0.02 (3)

### (I) Potassium trifluorido(4-methoxyphenyl)borate Special details

**Table:**

Geometry. All e.s.d.'s (except the e.s.d. in the dihedral angle between two l.s. planes) are estimated using the full covariance matrix. The cell e.s.d.'s are taken into account individually in the estimation of e.s.d.'s in distances, angles and torsion angles; correlations between e.s.d.'s in cell parameters are only used when they are defined by crystal symmetry. An approximate (isotropic) treatment of cell e.s.d.'s is used for estimating e.s.d.'s involving l.s. planes.
Refinement. Refinement of *F*^2^ against ALL reflections. The weighted *R*-factor *wR* and goodness of fit *S* are based on *F*^2^, conventional *R*-factors *R* are based on *F*, with *F* set to zero for negative *F*^2^. The threshold expression of *F*^2^ > σ(*F*^2^) is used only for calculating *R*-factors(gt) *etc*. and is not relevant to the choice of reflections for refinement. *R*-factors based on *F*^2^ are statistically about twice as large as those based on *F*, and *R*- factors based on ALL data will be even larger.

### (I) Potassium trifluorido(4-methoxyphenyl)borate Fractional atomic coordinates and isotropic or equivalent isotropic displacement parameters (Å^2^)

**Table:**

	*x*	*y*	*z*	*U*_iso_*/*U*_eq_	
K1	0.93636 (3)	0.577078 (16)	0.49116 (4)	0.01659 (9)	
C1	0.48663 (17)	0.69908 (7)	0.3989 (2)	0.0159 (3)	
C2	0.36313 (18)	0.72309 (8)	0.2628 (2)	0.0195 (3)	
H2	0.2718	0.6875	0.2199	0.023*	
C3	0.36757 (18)	0.79722 (8)	0.1867 (2)	0.0223 (3)	
H3	0.2813	0.8114	0.0938	0.027*	
C4	0.5004 (2)	0.84983 (8)	0.2493 (2)	0.0208 (3)	
C5	0.62439 (19)	0.82828 (8)	0.3880 (2)	0.0233 (3)	
H5	0.7139	0.8643	0.4324	0.028*	
C6	0.61673 (19)	0.75407 (8)	0.4608 (2)	0.0206 (3)	
H6	0.7018	0.7402	0.5549	0.025*	
C7	0.3970 (3)	0.94712 (11)	0.0422 (3)	0.0393 (5)	
H7A	0.4278	1.0000	0.0044	0.059*	
H7B	0.2674	0.9454	0.0865	0.059*	
H7C	0.4105	0.9121	−0.0622	0.059*	
B1	0.48502 (18)	0.61227 (8)	0.4743 (2)	0.0153 (3)	
F1	0.57562 (10)	0.55930 (5)	0.35081 (13)	0.01754 (18)	
F2	0.30347 (10)	0.58074 (4)	0.50165 (17)	0.02096 (19)	
F3	0.58402 (11)	0.60460 (5)	0.64078 (13)	0.02023 (19)	
O1	0.52053 (18)	0.92364 (6)	0.1842 (2)	0.0297 (3)	

### (I) Potassium trifluorido(4-methoxyphenyl)borate Atomic displacement parameters (Å^2^)

**Table:**

	*U*^11^	*U*^22^	*U*^33^	*U*^12^	*U*^13^	*U*^23^
K1	0.01225 (13)	0.02391 (15)	0.01361 (16)	0.00081 (8)	0.00031 (12)	0.00042 (12)
C1	0.0132 (5)	0.0193 (6)	0.0152 (6)	0.0009 (5)	0.0007 (5)	−0.0009 (5)
C2	0.0199 (6)	0.0204 (6)	0.0183 (7)	−0.0015 (5)	−0.0044 (5)	−0.0014 (6)
C3	0.0233 (6)	0.0229 (6)	0.0206 (7)	0.0011 (5)	−0.0043 (5)	0.0017 (6)
C4	0.0234 (6)	0.0167 (6)	0.0222 (7)	0.0007 (5)	0.0007 (6)	0.0011 (6)
C5	0.0223 (6)	0.0200 (6)	0.0277 (8)	−0.0036 (5)	−0.0054 (5)	−0.0028 (6)
C6	0.0181 (5)	0.0211 (6)	0.0226 (8)	0.0002 (5)	−0.0046 (5)	−0.0010 (6)
C7	0.0421 (9)	0.0273 (7)	0.0484 (13)	0.0011 (7)	−0.0085 (8)	0.0167 (9)
B1	0.0121 (5)	0.0185 (6)	0.0153 (7)	0.0000 (4)	0.0002 (6)	0.0008 (6)
F1	0.0182 (4)	0.0196 (4)	0.0148 (5)	0.0031 (3)	0.0025 (3)	−0.0007 (4)
F2	0.0129 (3)	0.0209 (3)	0.0291 (5)	−0.0015 (2)	0.0028 (4)	−0.0004 (3)
F3	0.0204 (3)	0.0271 (4)	0.0132 (5)	−0.0004 (3)	−0.0022 (3)	0.0025 (4)
O1	0.0352 (5)	0.0176 (5)	0.0362 (8)	−0.0009 (4)	−0.0037 (6)	0.0052 (5)

### (I) Potassium trifluorido(4-methoxyphenyl)borate Geometric parameters (Å, º)

**Table:**

K1—F3^i^	2.6156 (10)	C4—C5	1.398 (2)
K1—F2^ii^	2.6211 (7)	C5—C6	1.390 (2)
K1—F1^iii^	2.6550 (10)	C5—H5	0.9500
K1—F1^iv^	2.7568 (10)	C6—H6	0.9500
K1—F3	2.7836 (8)	C7—O1	1.423 (2)
K1—F1	2.7887 (8)	C7—H7A	0.9800
K1—F2^iv^	2.8885 (8)	C7—H7B	0.9800
K1—F3^iv^	3.4886 (9)	C7—H7C	0.9800
C1—C2	1.3940 (19)	B1—F3	1.4162 (19)
C1—C6	1.4036 (18)	B1—F2	1.4196 (14)
C1—B1	1.5987 (18)	B1—F1	1.4403 (17)
C2—C3	1.398 (2)	B1—K1^v^	3.2930 (14)
C2—K1^i^	3.5183 (15)	F1—K1^i^	2.6550 (10)
C2—H2	0.9500	F1—K1^v^	2.7568 (10)
C3—C4	1.3910 (19)	F2—K1^vi^	2.6211 (7)
C3—H3	0.9500	F2—K1^v^	2.8885 (8)
C4—O1	1.3696 (17)	F3—K1^iii^	2.6157 (10)
			
F3^i^—K1—F2^ii^	94.59 (3)	O1—C4—C5	115.78 (13)
F3^i^—K1—F1^iii^	173.67 (3)	C3—C4—C5	119.79 (13)
F2^ii^—K1—F1^iii^	90.34 (3)	C6—C5—C4	119.99 (13)
F3^i^—K1—F1^iv^	79.00 (3)	C6—C5—H5	120.0
F2^ii^—K1—F1^iv^	70.82 (2)	C4—C5—H5	120.0
F1^iii^—K1—F1^iv^	106.45 (2)	C5—C6—C1	121.78 (14)
F3^i^—K1—F3	107.78 (2)	C5—C6—H6	119.1
F2^ii^—K1—F3	152.47 (3)	C1—C6—H6	119.1
F1^iii^—K1—F3	66.40 (3)	O1—C7—H7A	109.5
F1^iv^—K1—F3	128.21 (2)	O1—C7—H7B	109.5
F3^i^—K1—F1	66.83 (3)	H7A—C7—H7B	109.5
F2^ii^—K1—F1	159.35 (3)	O1—C7—H7C	109.5
F1^iii^—K1—F1	108.89 (2)	H7A—C7—H7C	109.5
F1^iv^—K1—F1	95.79 (2)	H7B—C7—H7C	109.5
F3—K1—F1	47.98 (3)	F3—B1—F2	107.30 (13)
F3^i^—K1—F2^iv^	100.35 (3)	F3—B1—F1	104.95 (10)
F2^ii^—K1—F2^iv^	110.489 (19)	F2—B1—F1	104.73 (10)
F1^iii^—K1—F2^iv^	81.59 (3)	F3—B1—C1	112.45 (11)
F1^iv^—K1—F2^iv^	47.24 (3)	F2—B1—C1	114.56 (11)
F3—K1—F2^iv^	81.63 (2)	F1—B1—C1	112.10 (12)
F1—K1—F2^iv^	66.60 (3)	B1—F1—K1^i^	122.39 (8)
C2—C1—C6	116.59 (12)	B1—F1—K1^v^	98.47 (7)
C2—C1—B1	121.46 (12)	K1^i^—F1—K1^v^	117.24 (3)
C6—C1—B1	121.90 (12)	B1—F1—K1	96.46 (7)
C1—C2—C3	122.95 (12)	K1^i^—F1—K1	112.53 (3)
C1—C2—K1^i^	86.24 (8)	K1^v^—F1—K1	106.81 (3)
C3—C2—K1^i^	114.99 (10)	B1—F2—K1^vi^	156.48 (8)
C1—C2—H2	118.5	B1—F2—K1^v^	93.39 (6)
C3—C2—H2	118.5	K1^vi^—F2—K1^v^	107.73 (2)
K1^i^—C2—H2	68.1	B1—F3—K1^iii^	146.64 (8)
C4—C3—C2	118.88 (13)	B1—F3—K1	97.29 (7)
C4—C3—H3	120.6	K1^iii^—F3—K1	113.95 (3)
C2—C3—H3	120.6	C4—O1—C7	117.09 (13)
O1—C4—C3	124.43 (14)		
			
C6—C1—C2—C3	1.2 (2)	C2—C1—C6—C5	−1.1 (2)
B1—C1—C2—C3	−176.35 (14)	B1—C1—C6—C5	176.46 (14)
C6—C1—C2—K1^i^	118.72 (12)	C2—C1—B1—F3	−164.66 (12)
B1—C1—C2—K1^i^	−58.84 (12)	C6—C1—B1—F3	17.90 (18)
C1—C2—C3—C4	−0.2 (2)	C2—C1—B1—F2	−41.81 (19)
K1^i^—C2—C3—C4	−102.69 (14)	C6—C1—B1—F2	140.76 (15)
C2—C3—C4—O1	179.10 (14)	C2—C1—B1—F1	77.36 (16)
C2—C3—C4—C5	−0.9 (2)	C6—C1—B1—F1	−100.07 (15)
O1—C4—C5—C6	−178.99 (14)	C3—C4—O1—C7	−0.4 (2)
C3—C4—C5—C6	1.0 (2)	C5—C4—O1—C7	179.62 (15)
C4—C5—C6—C1	0.0 (2)		

Symmetry codes: (i) −*x*+3/2, *y*, *z*−1/2; (ii) *x*+1, *y*, *z*; (iii) −*x*+3/2, *y*, *z*+1/2; (iv) *x*+1/2, −*y*+1, *z*; (v) *x*−1/2, −*y*+1, *z*; (vi) *x*−1, *y*, *z*.

### (I) Potassium trifluorido(4-methoxyphenyl)borate Hydrogen-bond geometry (Å, º)

Cg1 is the centroid of the C1–C6 benzene ring.

**Table:**

*D*—H···*A*	*D*—H	H···*A*	*D*···*A*	*D*—H···*A*
C2—H2···F2^vii^	0.95	2.50	3.3359 (17)	147
C7—H7a···O1^viii^	0.98	2.72	3.496 (1)	137
C3—H3···*Cg*1^vii^	0.95	2.85	3.7171 (15)	152

Symmetry codes: (vii) −*x*+1/2, *y*, *z*−1/2; (viii) −*x*+1, −*y*+2, *z*−1/2.

### (II) Potassium trifluorido(4-fluorophenyl)borate Crystal data

**Table:**

K^+^·C_6_H_4_BF_4_^−^	*F*(000) = 800
*M**_r_* = 202.00	*D*_x_ = 1.751 Mg m^−^^3^
Orthorhombic, *P**b**c**a*	Mo *K*α radiation, λ = 0.71075 Å
Hall symbol: -P 2ac 2ab	Cell parameters from 8210 reflections
*a* = 7.1317 (5) Å	θ = 3.2–27.5°
*b* = 7.3757 (5) Å	µ = 0.70 mm^−^^1^
*c* = 29.129 (2) Å	*T* = 100 K
*V* = 1532.22 (18) Å^3^	Block, colourless
*Z* = 8	0.07 × 0.05 × 0.01 mm

### (II) Potassium trifluorido(4-fluorophenyl)borate Data collection

**Table:**

Rigaku CCD diffractometer	1726 independent reflections
Radiation source: fine-focus sealed tube	1435 reflections with *I* > 2σ(*I*)
Graphite monochromator	*R*_int_ = 0.037
ω scans	θ_max_ = 27.5°, θ_min_ = 3.2°
Absorption correction: multi-scan (*SADABS*; Bruker, 2012)	*h* = −9→8
*T*_min_ = 0.953, *T*_max_ = 0.993	*k* = −9→7
9537 measured reflections	*l* = −32→37

### (II) Potassium trifluorido(4-fluorophenyl)borate Refinement

**Table:**

Refinement on *F*^2^	Primary atom site location: structure-invariant direct methods
Least-squares matrix: full	Secondary atom site location: difference Fourier map
*R*[*F*^2^ > 2σ(*F*^2^)] = 0.027	Hydrogen site location: inferred from neighbouring sites
*wR*(*F*^2^) = 0.065	H-atom parameters constrained
*S* = 1.06	*w* = 1/[σ^2^(*F*_o_^2^) + (0.0274*P*)^2^ + 0.6658*P*] where *P* = (*F*_o_^2^ + 2*F*_c_^2^)/3
1726 reflections	(Δ/σ)_max_ = 0.001
109 parameters	Δρ_max_ = 0.27 e Å^−^^3^
0 restraints	Δρ_min_ = −0.23 e Å^−^^3^

### (II) Potassium trifluorido(4-fluorophenyl)borate Special details

**Table:**

Geometry. All e.s.d.'s (except the e.s.d. in the dihedral angle between two l.s. planes) are estimated using the full covariance matrix. The cell e.s.d.'s are taken into account individually in the estimation of e.s.d.'s in distances, angles and torsion angles; correlations between e.s.d.'s in cell parameters are only used when they are defined by crystal symmetry. An approximate (isotropic) treatment of cell e.s.d.'s is used for estimating e.s.d.'s involving l.s. planes.
Refinement. Refinement of *F*^2^ against ALL reflections. The weighted *R*-factor *wR* and goodness of fit *S* are based on *F*^2^, conventional *R*-factors *R* are based on *F*, with *F* set to zero for negative *F*^2^. The threshold expression of *F*^2^ > σ(*F*^2^) is used only for calculating *R*-factors(gt) *etc*. and is not relevant to the choice of reflections for refinement. *R*-factors based on *F*^2^ are statistically about twice as large as those based on *F*, and *R*- factors based on ALL data will be even larger.

### (II) Potassium trifluorido(4-fluorophenyl)borate Fractional atomic coordinates and isotropic or equivalent isotropic displacement parameters (Å^2^)

**Table:**

	*x*	*y*	*z*	*U*_iso_*/*U*_eq_	
K1	0.06924 (4)	0.02356 (5)	0.205013 (12)	0.01668 (10)	
C1	0.5118 (2)	0.1023 (2)	0.13156 (5)	0.0161 (3)	
C2	0.3892 (2)	0.0245 (2)	0.09978 (6)	0.0213 (3)	
H2	0.3130	−0.0745	0.1091	0.026*	
C3	0.3750 (2)	0.0869 (2)	0.05506 (6)	0.0245 (4)	
H3	0.2905	0.0324	0.0339	0.029*	
C4	0.4867 (2)	0.2301 (2)	0.04213 (5)	0.0236 (4)	
C5	0.6112 (2)	0.3121 (2)	0.07145 (6)	0.0250 (4)	
H5	0.6871	0.4105	0.0616	0.030*	
C6	0.6226 (2)	0.2463 (2)	0.11604 (6)	0.0207 (3)	
H6	0.7085	0.3011	0.1367	0.025*	
B1	0.5200 (2)	0.0373 (2)	0.18355 (6)	0.0147 (3)	
F1	0.43225 (11)	0.16564 (12)	0.21396 (3)	0.0178 (2)	
F2	0.70288 (11)	0.01321 (13)	0.20154 (3)	0.0210 (2)	
F3	0.42107 (11)	−0.12680 (12)	0.19106 (3)	0.0193 (2)	
F4	0.47218 (15)	0.29343 (14)	−0.00157 (3)	0.0327 (3)	

### (II) Potassium trifluorido(4-fluorophenyl)borate Atomic displacement parameters (Å^2^)

**Table:**

	*U*^11^	*U*^22^	*U*^33^	*U*^12^	*U*^13^	*U*^23^
K1	0.01228 (15)	0.01386 (17)	0.02390 (18)	0.00022 (12)	0.00079 (12)	0.00020 (13)
C1	0.0143 (6)	0.0139 (7)	0.0200 (8)	0.0019 (6)	0.0007 (6)	−0.0006 (6)
C2	0.0214 (7)	0.0201 (8)	0.0225 (8)	−0.0029 (7)	−0.0016 (6)	0.0002 (7)
C3	0.0268 (8)	0.0256 (9)	0.0211 (9)	−0.0019 (7)	−0.0056 (7)	−0.0016 (7)
C4	0.0306 (9)	0.0243 (9)	0.0160 (8)	0.0046 (7)	0.0013 (6)	0.0020 (7)
C5	0.0296 (9)	0.0221 (9)	0.0234 (9)	−0.0056 (7)	0.0052 (6)	0.0014 (7)
C6	0.0222 (7)	0.0189 (8)	0.0211 (8)	−0.0050 (7)	0.0007 (6)	−0.0014 (7)
B1	0.0116 (7)	0.0130 (8)	0.0194 (8)	0.0014 (6)	0.0006 (6)	−0.0008 (6)
F1	0.0184 (4)	0.0152 (5)	0.0198 (5)	0.0027 (4)	0.0027 (3)	−0.0012 (4)
F2	0.0125 (4)	0.0295 (5)	0.0209 (5)	0.0032 (4)	−0.0024 (3)	−0.0012 (4)
F3	0.0205 (4)	0.0125 (5)	0.0247 (5)	−0.0017 (4)	0.0003 (4)	0.0026 (4)
F4	0.0474 (6)	0.0332 (6)	0.0174 (5)	−0.0013 (5)	−0.0007 (5)	0.0061 (4)

### (II) Potassium trifluorido(4-fluorophenyl)borate Geometric parameters (Å, º)

**Table:**

K1—F3^i^	2.6116 (10)	C4—F4	1.3597 (18)
K1—F2^ii^	2.6159 (9)	C4—C5	1.373 (2)
K1—F1^iii^	2.6527 (9)	C5—C6	1.389 (2)
K1—F1^iv^	2.7612 (10)	C5—H5	0.9500
K1—F3	2.7732 (9)	C6—H6	0.9500
K1—F1	2.8050 (9)	B1—F2	1.4166 (17)
K1—F2^iv^	2.8853 (10)	B1—F3	1.4182 (19)
K1—F3^iv^	3.3927 (10)	B1—F1	1.4393 (18)
C1—C2	1.397 (2)	B1—K1^v^	3.2665 (18)
C1—C6	1.399 (2)	F1—K1^i^	2.6527 (9)
C1—B1	1.590 (2)	F1—K1^v^	2.7612 (9)
C2—C3	1.385 (2)	F2—K1^vi^	2.6159 (9)
C2—H2	0.9500	F2—K1^v^	2.8852 (10)
C3—C4	1.375 (2)	F3—K1^iii^	2.6116 (10)
C3—H3	0.9500	F3—K1^v^	3.3928 (10)
			
F3^i^—K1—F2^ii^	92.82 (3)	F4—C4—C3	118.44 (15)
F3^i^—K1—F1^iii^	176.44 (3)	C5—C4—C3	122.87 (16)
F2^ii^—K1—F1^iii^	88.32 (3)	C4—C5—C6	117.74 (16)
F3^i^—K1—F1^iv^	76.56 (3)	C4—C5—H5	121.1
F2^ii^—K1—F1^iv^	71.99 (3)	C6—C5—H5	121.1
F1^iii^—K1—F1^iv^	107.00 (2)	C5—C6—C1	122.34 (15)
F3^i^—K1—F3	110.37 (2)	C5—C6—H6	118.8
F2^ii^—K1—F3	152.42 (3)	C1—C6—H6	118.8
F1^iii^—K1—F3	67.67 (3)	F2—B1—F3	107.08 (12)
F1^iv^—K1—F3	126.67 (3)	F2—B1—F1	104.80 (12)
F3^i^—K1—F1	67.74 (3)	F3—B1—F1	104.48 (11)
F2^ii^—K1—F1	159.45 (3)	F2—B1—C1	115.08 (12)
F1^iii^—K1—F1	111.49 (2)	F3—B1—C1	112.70 (13)
F1^iv^—K1—F1	96.05 (2)	F1—B1—C1	111.86 (12)
F3—K1—F1	47.78 (3)	F2—B1—K1^v^	61.95 (7)
F3^i^—K1—F2^iv^	99.45 (3)	F3—B1—K1^v^	82.74 (8)
F2^ii^—K1—F2^iv^	111.45 (2)	F1—B1—K1^v^	57.03 (7)
F1^iii^—K1—F2^iv^	83.24 (3)	C1—B1—K1^v^	163.77 (11)
F1^iv^—K1—F2^iv^	47.18 (2)	F2—B1—K1	145.97 (10)
F3—K1—F2^iv^	80.14 (3)	F3—B1—K1	57.04 (6)
F1—K1—F2^iv^	67.51 (3)	F1—B1—K1	58.42 (6)
F3^i^—K1—F3^iv^	118.03 (3)	C1—B1—K1	98.93 (9)
F2^ii^—K1—F3^iv^	73.38 (3)	K1^v^—B1—K1	85.12 (4)
F1^iii^—K1—F3^iv^	65.53 (2)	B1—F1—K1^i^	126.56 (8)
F1^iv^—K1—F3^iv^	41.51 (2)	B1—F1—K1^v^	97.04 (8)
F3—K1—F3^iv^	106.37 (3)	K1^i^—F1—K1^v^	117.58 (3)
F1—K1—F3^iv^	109.07 (3)	B1—F1—K1	95.66 (8)
F2^iv^—K1—F3^iv^	41.62 (2)	K1^i^—F1—K1	111.04 (3)
C2—C1—C6	116.82 (15)	K1^v^—F1—K1	105.32 (3)
C2—C1—B1	122.05 (14)	B1—F2—K1^vi^	158.46 (9)
C6—C1—B1	121.08 (14)	B1—F2—K1^v^	92.38 (8)
C3—C2—C1	122.18 (15)	K1^vi^—F2—K1^v^	107.01 (3)
C3—C2—H2	118.9	B1—F3—K1^iii^	148.62 (8)
C1—C2—H2	118.9	B1—F3—K1	97.55 (8)
C4—C3—C2	118.06 (15)	K1^iii^—F3—K1	113.33 (3)
C4—C3—H3	121.0	B1—F3—K1^v^	72.76 (8)
C2—C3—H3	121.0	K1^iii^—F3—K1^v^	100.12 (3)
F4—C4—C5	118.69 (15)	K1—F3—K1^v^	91.16 (3)
			
C6—C1—C2—C3	−0.7 (2)	C2—C1—C6—C5	0.8 (2)
B1—C1—C2—C3	176.65 (15)	B1—C1—C6—C5	−176.56 (15)
C1—C2—C3—C4	0.1 (3)	C2—C1—B1—F2	135.24 (15)
C2—C3—C4—F4	−179.32 (15)	C6—C1—B1—F2	−47.6 (2)
C2—C3—C4—C5	0.4 (3)	C2—C1—B1—F3	12.0 (2)
F4—C4—C5—C6	179.43 (15)	C6—C1—B1—F3	−170.75 (13)
C3—C4—C5—C6	−0.3 (3)	C2—C1—B1—F1	−105.32 (17)
C4—C5—C6—C1	−0.3 (3)	C6—C1—B1—F1	71.88 (18)

Symmetry codes: (i) −*x*+1/2, *y*+1/2, *z*; (ii) *x*−1, *y*, *z*; (iii) −*x*+1/2, *y*−1/2, *z*; (iv) *x*−1/2, *y*, −*z*+1/2; (v) *x*+1/2, *y*, −*z*+1/2; (vi) *x*+1, *y*, *z*.

### (II) Potassium trifluorido(4-fluorophenyl)borate Hydrogen-bond geometry (Å, º)

**Table:**

*D*—H···*A*	*D*—H	H···*A*	*D*···*A*	*D*—H···*A*
C6—H6···F2^vii^	0.95	2.53	3.4099 (19)	154

Symmetry code: (vii) −*x*+3/2, *y*+1/2, *z*.
